# Whole genome detection of sequence and structural polymorphism in six diverse horses

**DOI:** 10.1371/journal.pone.0230899

**Published:** 2020-04-09

**Authors:** Mohammed Ali Al Abri, Heather Marie Holl, Sara E. Kalla, Nathan B. Sutter, Samantha A. Brooks

**Affiliations:** 1 Department of Animal and Veterinary Sciences, College of Agriculture and Marine Sciences, Sultan Qaboos University, Al Khod, Muscat, Oman; 2 Department of Animal Science, Cornell University, Ithaca, NY, United States of America; 3 Department of Clinical Sciences, College of Veterinary Medicine, Cornell University, Ithaca, NY, United States of America; 4 Department of Biology, La Sierra University, Riverwalk Parkway, Riverside, CA, United States of America; 5 Department of Animal Sciences, University of Florida Genetics Institute, University of Florida, Gainesville, FL, United States of America; Universite de Lausanne Faculte de biologie et medecine, SWITZERLAND

## Abstract

The domesticated horse has played a unique role in human history, serving not just as a source of animal protein, but also as a catalyst for long-distance migration and military conquest. As a result, the horse developed unique physiological adaptations to meet the demands of both their climatic environment and their relationship with man. Completed in 2009, the first domesticated horse reference genome assembly (EquCab 2.0) produced most of the publicly available genetic variations annotations in this species. Yet, there are around 400 geographically and physiologically diverse breeds of horse. To enrich the current collection of genetic variants in the horse, we sequenced whole genomes from six horses of six different breeds: an American Miniature, a Percheron, an Arabian, a Mangalarga Marchador, a Native Mongolian Chakouyi, and a Tennessee Walking Horse, and mapped them to EquCab3.0 genome. Aside from extreme contrasts in body size, these breeds originate from diverse global locations and each possess unique adaptive physiology. A total of 1.3 billion reads were generated for the six horses with coverage between 15x to 24x per horse. After applying rigorous filtration, we identified and functionally annotated 17,514,723 Single Nucleotide Polymorphisms (SNPs), and 1,923,693 Insertions/Deletions (INDELs), as well as an average of 1,540 Copy Number Variations (CNVs) and 3,321 Structural Variations (SVs) per horse. Our results revealed putative functional variants including genes associated with size variation like *LCORL* gene (found in all horses), *ZFAT* in the Arabian, American Miniature and Percheron horses and *ANKRD1* in the Native Mongolian Chakouyi horse. We detected a copy number variation in the *Latherin* gene that may be the result of evolutionary selection impacting thermoregulation by sweating, an important component of athleticism and heat tolerance. The newly discovered variants were formatted into user-friendly browser tracks and will provide a foundational database for future studies of the genetic underpinnings of diverse phenotypes within the horse.

## Author summary

The domesticated horse played a unique role in human history, serving not just as a source of dietary animal protein, but also as a catalyst for long-distance migration and military conquest. As a result, the horse developed unique physiological adaptations to meet the demands of both their climatic environment and their relationship with man. Although the completion of the horse reference genome allowed for the discovery of many genetic variants, the remarkable diversity across breeds of horse calls for additional effort to quantify the complete span of genetic polymorphism within this unique species. In this work, we present genome re-sequencing and variant detection analysis for six horses belonging to six different breeds representing different morphology, origins and vary in their physiological demands and response. We identified and annotated not just single nucleotide polymorphisms (SNPs), but also insertions and deletions (INDELs), copy number variations (CNVs) and structural variations (SVs). Our results illustrate novel sources of polymorphism and highlight potentially impactful variations for phenotypes of body size and conformation. We also detected a copy number loss in the *Latherin* gene that could be the result of an evolutionary selection affecting thermoregulation through sweating. Our newly discovered variants were formatted into easy-to-use tracks that can be easily accessed by researchers around the globe.

## Introduction

Quantifying genetic variation is an important theme in modern biology and population genetics. Recent technological advances in genomics have benefitted livestock by allowing examination of genetic variation in these non-traditional model species at an unprecedented scale and resolution. Cataloging that variation lays the foundation for dissecting the complex genetic architecture of phenotypic variation, which in turn has many applications in livestock health, welfare, physiology and production traits [1,2]. Inferring these variations also improves our current understanding of ancient demographic and evolutionary histories, as well as the mechanisms underlying adaptation in various species [3]. In addition, cross-species comparisons of genetic variation improves our understanding of the structure-to-function relationship within conserved elements of the mammalian genome [4].

Domesticated approximately 5,500 years ago, horses were historically used for agriculture, transportation, trade, warfare, and as draught animals [5]. Man has since selected horses suitable for a range of physical and behaviorally desirable traits, ultimately resulting in the formation of more than 400 unique horse breeds [6]. Comparisons between ancient and domesticated horse genomes revealed signatures of this selective pressure in ~125 potential domestication target genes [5]. Advantageously, the *Equidae* possess a particularly old and diverse fossil record, aiding not only in characterizing their demographic history but also ancient human movement and migration [3,5,6]. However, compared to other livestock species, relatively few studies have focused on the discovery of the standing genetic variation within different horse breeds [7]. Therefore, additional investigation of the equine genomic architecture is critical for a better understanding of the equine genome, as well as for expanded comparisons across diverse mammalian species. Furthermore, the equine industry itself provides an eager opportunity to apply genomic discoveries towards improvements in the health and well-being of this valuable livestock species.

Here, we sequenced six horses belonging to six divergent breeds (one horse from each breed) in order to enrich the current collection of genetic variants in the horse. Namely, we sequenced a female Percheron (PER) and an American Miniature (AMH), and an Arabian (ARB), a Tennessee Walking Horse (TWH), a Mangalarga Marchador (MM) and a Native Mongolian Chakouyi (CH) male horses. These breeds were historically selected to perform distinct tasks and therefore may harbor a wealth of unique variation at the genome level. For example, the Arabian horse was primarily selected for metabolic efficiency, endurance and strength. Severe desert conditions required a resilient animal with absolute loyalty. Arabian horses were central to the survival and culture of the Bedouins peoples of the Arabian Peninsula [8]. The Percheron horse was primarily selected for large size and developed as draft horse and was used as both a war horse and a farm horse [9]. On the other hand, the American miniature horse were selected for small body size [10], whereas the Native Mongolian Chakouyi was selected for its unique smooth gait [11,12]. The Mangalarga Marchador is a Brazilian Iberian horse breed that was selected for its versatility and stamina. It is capable of performing diverse tasks, from performance racing to herding cattle [13]. The Tennessee Walking Horse (or Tennessee Walker) is a unique in that it is the only U.S. breed able to perform an even-timed 4-beat gait called the “running-walk” at intermediate speeds [14].

Our goal was to investigate not just single nucleotide polymorphisms (SNPs) and small insertion deletions (INDELs), but also copy number variations (CNVs) and structural variations (SV). After quality filtering of the reads, we detected and annotated the variation in each of these four classes within the six horses. Raw SNPs and INDELs are now available in European Nucleotide Archive (ENA). On the other hand annotated SNPs and INDELs as well as CNVs and SVs (which are often difficult to access) in public databases have processed into user-friendly tracks which were deposited at CyVerse (https://data.cyverse.org/).

## Results and discussion

### Whole genome sequencing, alignment and quality control

High throughput Next Generation DNA Sequencing (NGS) provides affordable access to study genome wide genetic variation. We used paired-end Illumina sequencing to interrogate the genomes of six horses from six different breeds to an average sequence coverage of 10x to 24x. Namely, these were an Arabian, a Percheron (a breed of primarily French origin), an American Miniature, a Mangalarga Marchador (from Brazil), a Native Mongolian Chakouyi, and a Tennessee Walking Horse. Sequencing was done using the Illumina HiSeq2500 (Illumina, San Diego, CA) with manufacturer recommended reagents and procedures by the Biotechnology Resource Center at Cornell University. A total of 1.3 billion reads were generated for the six horses. The raw number of reads ranged between 142 million reads on the Native Mongolian horse to 324 million reads on the American miniature (**Table 1**). After filtering, between 121 million (Native Mongolian horse) and 187 million (Percheron) paired reads were retained. Both read pairs aligned to the EquCab3.0 reference genome [1] in 99% of reads of all horses, indicating a successful mapping procedure (**Table 1**).

**Table 1:** Yield, filtering and mapping summary of the next generation sequencing data of six horses from different breeds. The depth of coverage and mapping metrics show a descent quality of the 6 genomes sequencing and genome coverage.

	ARB	PER	AMH	TWH	MM	CH
Number of paired-end reads before trimming	241,480,555	296,460,133	324,123,384	198,749,393	169,680,137	142,502,233
Read lengths	100/100	100/100	100/100	150/150	150/150	150/150
Estimated average depth of coverage before trimming^1^	17.8x	21.96x	24x	22.0x	18.9x	15.833x
Number of paired-end reads after trimming	165,277,009	187,223,705	138,772,441	161,659,278	134,732,394	121,744,242
Total number of aligned reads^1^	330,554,018	374,447,410	277,544,882	323,318,556	269,464,788	243,488,484
Estimated depth of coverage ^2^	13.35619062	15.12972	11.21433	19.59576	16.33178	14.7574
Percentage of mapped reads^3^	99.8%	99.85%	99.7%	99.95%	99%	99.14%
Percentage of reads where both pairs mapped^3^	99.83%	99.84%	99.71%	99.54%	99.01%	99.12%

1 Calculated by multiplying the number of paired end reads after trimming by 2

2 Estimated from the total number of aligned reads using the formula C = L*N/G (where G is the haploid genome length (2,474,912,402), L is the read length and N is the number of reads).

3 Estimated using the bamtools *stats* procedure in bamtools.

### Identification of variants

#### SNPs

In total, 17,594,817 SNPs were detected using the GATK *HaplotypeCaller*. The number of SNPs is about 0.7% of the size of the genome (about 1 every 136 base pairs). Amongst those, 151,739 SNPs (0.8%) were multi-allelic. The mean transition to transversion ratio in these horses is 1.83 (range 1.74 to 1.93) (**Table 2**), a value very close to other mammalian species [15]. The mean, median and standard deviation of Phred-scaled quality scores for the SNPs were 585, 235 and 1,529 respectively, which signifies a very high call accuracy.

**Table 2:** Genotype categories of SNPs and INDELs and counts of CNVs and SVs in the six horses.

	ARB	PER	AMH	TWH	MM	CH
**SNPs**						
Homozygous Reference	11,555,233	10,905,683	10,908,015	10,795,424	10,052,722	10,259,979
Heterozygous	3,741,846	4,264,207	4,182,897	4,631,063	4,824,665	4,872,714
Homozygous Alternative	1,936,702	2,102,585	2,067,904	1,936,150	2,096,698	2,089,121
Missing	280,942	242,248	355,907	152,086	540,638	292,909
Transitions	4,936,199	5,473,518	5,298,618	5,331,846	5,801,750	5,662,053
Transversions	2,563,411	2,871,410	2,903,626	3,039,726	3,089,634	3,253,897
**INDELs**						
Homozygous Reference	1,289,788	1,256,004	1,283,913	1,106,243	1,039,000	1,127,564
Heterozygous	361,414	380,707	340,107	547,182	554,852	507,378
Homozygous Alternative	217,931	230,637	217,597	238,297	235,883	248,055
Missing	54,560	56,345	82,076	31,971	93,958	40,696
**CNVs**						
Gains	825	727	671	694	695	719
Losses	837	882	853	800	795	747
**Structural Variations**						
Interchromosomal	1,298	1,644	964	1,366	686	2,140
Intrachromosomal	2,986	2,336	1,696	1,750	928	263

**Fig 1:**
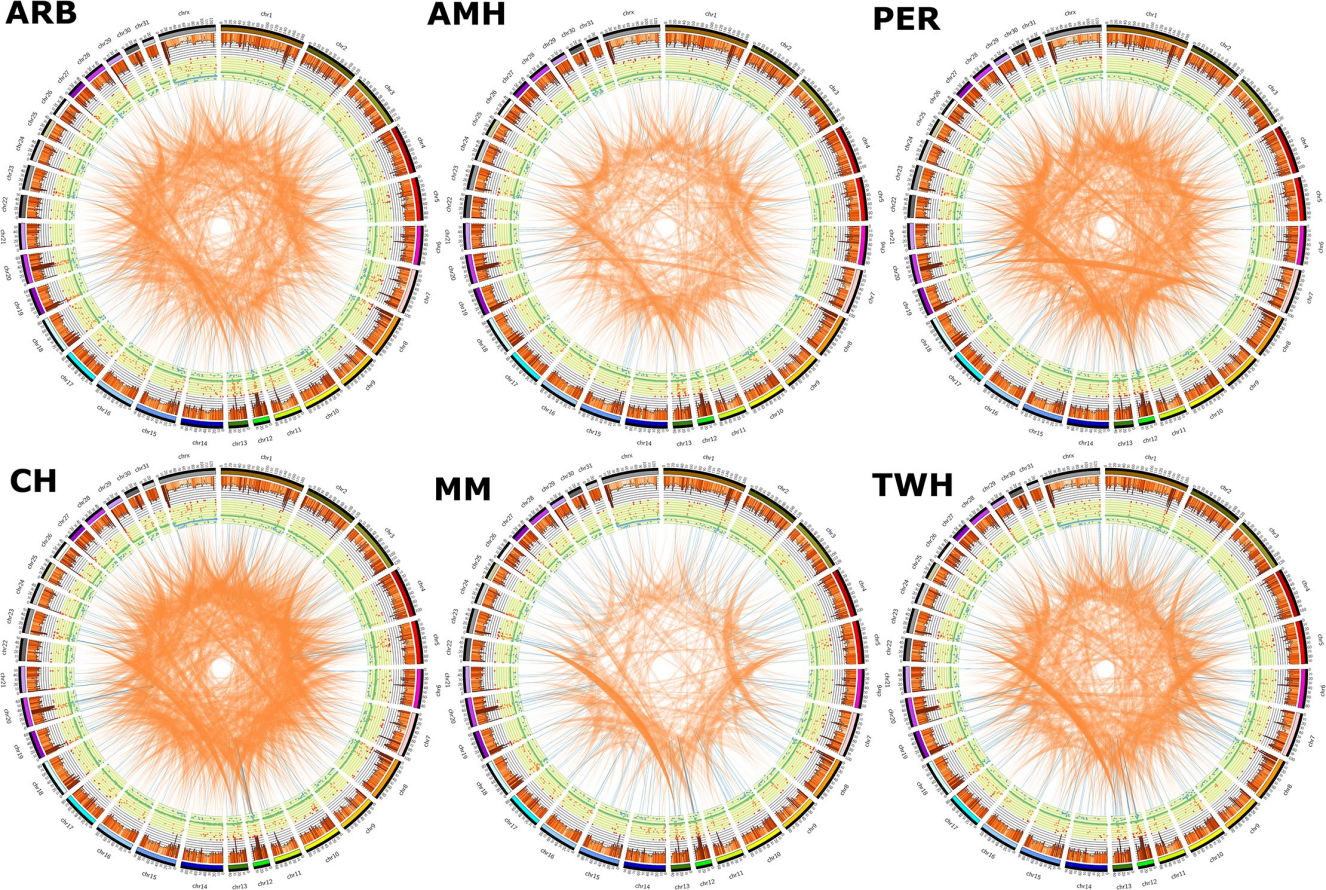
Circos plot summarizing the genetic variants detected in each horse. The pattern of variation across the genomes of the six horses reveals structurally diverse regions important for immunity and olfactory reception at chromosomes 12 and 20 in all six horses. From the inside out, each plot shows two endpoints of the inter- (orange) and intra- (blue) chromosomal translocations. Intrachromosomal translocations > 5 MB are in dark blue. The yellow ring shows the copy number variations (green = normal, blue = loss, red = gain). The histogram (in orange) shows the density of SNPs detected using 1MB windows.

The highest proportion of genotype calls were the homozygous reference genotype, comprising 58% to 67% of the SNPs in each horse (**Table 2**). Relative to the chromosome size, the highest proportion of SNPs was found in chromosome 12 (1%) followed by chromosome 20 (0.98%) (**Fig 1**). Overall, the highest proportion of homozygous reference genotypes was found in the Arabian horse. This may be explained by the fact that, among the breeds included, the Arabian horse has the closest historical relationship to the reference genome derived from a mare of the Thoroughbred breed. The Thoroughbred horse population originated by mating three prominent Arabian stallions to native mares in England during the 17^th^ century [16].

#### INDELs

It is well established that INDELs are the second most common form of genomic variations, altering a similar total proportion of base pairs as SNPs [17]. In horses, some known INDELs cause genetic disorders such as the Lavender Foal Syndrome (LFS) [18] and the Severe Combined Immunodeficiency (SCID) [19]. We detected 1,923,693 small INDEL loci using the GATK *HaplotypeCaller* procedure. Within this set, 10,811 INDELs were multi-allelic. The mean, median and standard deviation of Phred-scaled quality scores for the INDELs were 549, 184 and 1,770 respectively, which shows a lower accuracy and dispersion compared to that observed in SNPs. INDEL size ranged from 1 to 281bp, with mean of 1.5 bp and a median of 1 bp. The INDELs were more insertions than deletions (66% and 34% respectively), unlike what was observed in the INDELs pattern in humans [20], [21]. Similar to the SNPs, the most frequent small INDELs calls were the homozygous alternative calls, ranging between 54 and 57% of the total INDELs calls in each horse (**Table 2**). INDELs are more rare events than SNPs and are thus more likely to be unique to a breed of horses than to be shared between breeds [22]. In fact, the resolution of Eukaryotic phylogenetic trees can be improved by incorporating INDELs [23].

It is noteworthy that the incidence of homozygous alternative (non-reference homozygous) SNPs was consistently high in all horses. This could be a result of a greater divergence between the reference genome (a Thoroughbred horse) relative to the other breeds. The SNP and INDEL genotype missingness was inversely correlated with the estimated depth of coverage (-0.30 and -0.45 consecutively) and was the highest in the Mangalarga Marchadore (**Tables 1 and 2**). The total number of INDELs called within each genome was influenced by the depth of coverage, as previously observed in NGS data of human genomes [24].

#### CNVs and SVs

CNVs and SVs contribute significantly to genomic diversity [25]. Genome-wide datasets produced by NGS technologies are revealing a wealth of knowledge about the frequency and structure of these types of polymorphisms. Of the identified CNVs, the number of gains was comparable to the number of losses for all horses (**Table 2**, 721 mean gains vs 819 mean losses). 1887 of the CNVs detected in this study overlapped fully with CNVs reported earlier in a recent study [26] (**S7 Table**). Since many of the gains and losses are shared between horses, we hypothesize that those are artifacts of the computational assembly of EquCab3.0, compressing regions of repetitive sequences and highly homologous gene families. Numerous CNV regions (or genes) in the genome could represent duplication events incorrectly assigned to regions of high homology in EquCab3.0.

Additionally, we also observe a consistent excess of intrachromosomal SVs compared to the interchromosomal SVs (**Table 2**). Bias towards intrachromosomal SVs is not uncommon in this type of analysis and is often due to a preference for intrachromosomal joining resulting from the relative closer proximity of these genomic regions. This same phenomenon was observed in studies of the mouse [27], human [28] and chicken [29]. It is proposed that a biological mechanism preferring proximal intrachromosomal rearrangement reduces large-scale genomic alterations, and therefore maintains genomic stability [27].

The Arabian horse possessed the highest number of the Intrachromosomal SVs (n = 2,869) compared to other horses. This is approximately 1000 SVs higher than average of the corresponding values in the other horses and may be an artifact of imperfect library preparation or fragment size selection prior to sequencing. Indeed, filtering of such artifacts is a significant challenge for reliable discovery and genotyping of SVs. Notably, the highest number of interchromosomal SVs were detected in the Native Mongolian horse (n = 2,140) followed by the Percheron (n = 1,644).

#### Annotation of detected variants

The majority of SNPs were intergenic, followed by intronic, comprising 60% and 27% of SNPs, respectively (**S1 Table**) [30]. The small percentage of exonic SNPs (1.4%) and INDELs (0.69%) is likely the result of strong negative selective pressure exerted on coding regions due to the potentially severe functional implications of these alterations [21]. Likewise, a lower diversity around 3’ UTR and 5’ UTR regions was found in SNPs and INDELs (0.08% - 0.23%), which was also reported in other mammalian species [21,30]. The highest concentration of SNPs in the genomes of all the horses was observed amidst ECA12 (Equine chromosome 12) and ECA20 which had a rate of 1 SNP every 97 and 101 consecutively compared to a mean of (144) for all chromosomes. Functional annotation of these regions using PANTHER showed that they are involved in metabolic and sensory perception (ECA12) and immune response and antigen processing (ECA20). This could be indicative of an evolution of these genes. However, it might be the result of mis-assembles in the reference genome or misalignment of reads.

Copy number and structural variations are given relatively little attention compared to SNPs in studies of genetic diversity. Nevertheless, they are ubiquitous in the mammalian genome and influence a number of phenotypes [31,32]. The resulting CNVs and SVs were annotated with Bedtools (v2.23.0) using the UCSC genome browser xenoRefGene genes they overlap with (**S2 Table**). We found that chromosomes ECA1 possessed the highest density of CNVs possibly because it is the largest chromosome (**S3 Table**).

**Fig 2:**
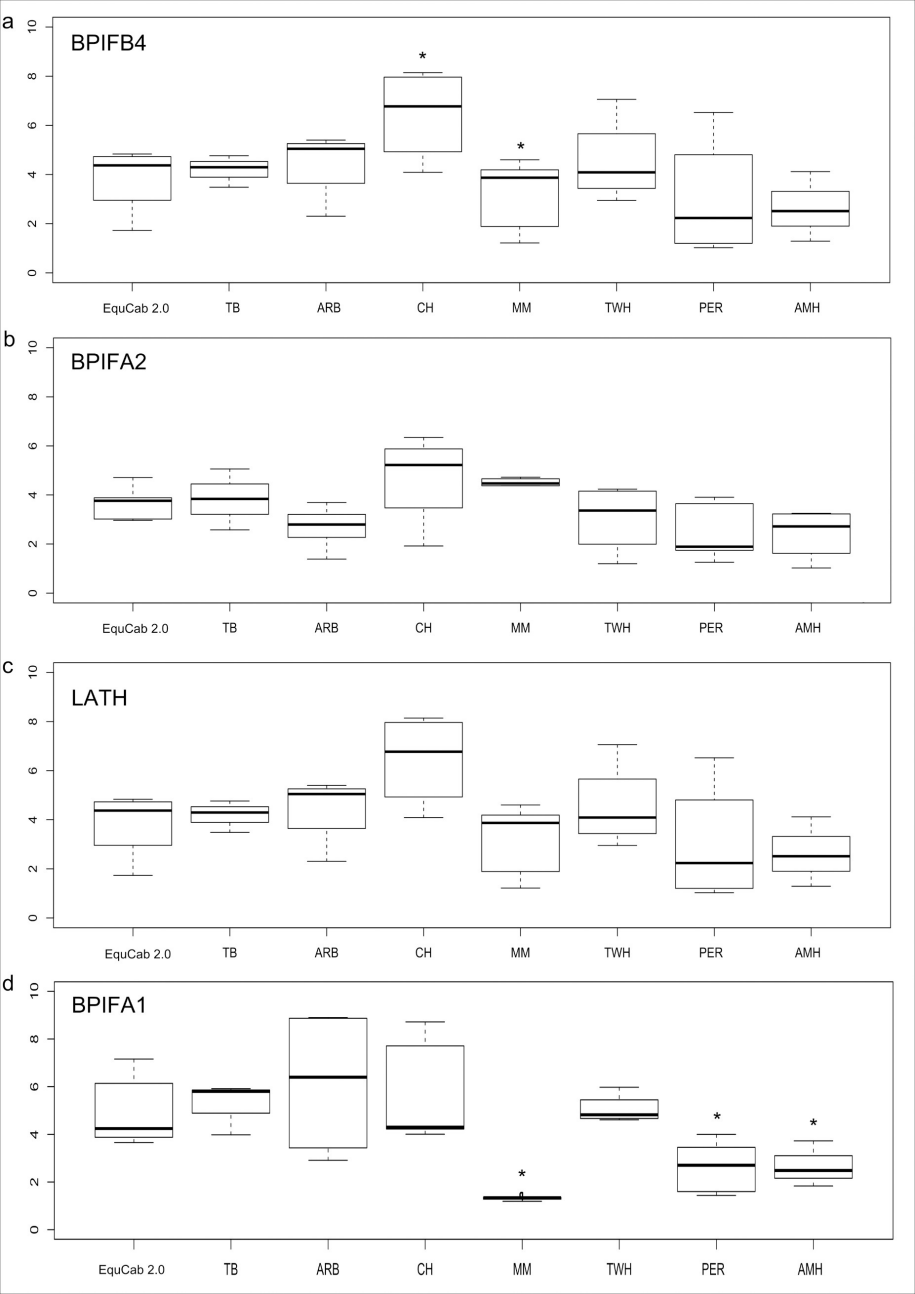
RT-qPCR results of the *LATH* CNV region for seven horses belonging to different breeds, a thoroughbred (TB), a Percheron (PER) and an American Miniature (AMH), and an Arabian (ARB), a Tennessee Walking Horse (TWH), a Mangalarga Marchador (MM) and a Native Mongolian Chakouyi (CH). The Y axis represents the copy number and the X axis, represents horses from different breeds. The results are shown for different primers in the order they appear in in the genome are shown, starting with *BPIFB4* to *BPIFA1*. The results show statistically significant difference in copy number variation between horses for *BPIFB4* (a) and *BPIFA1* (d) two genes that flank *LATH* (c) in the EquCab 2.0 assembly.

Our functional CNVs annotation revealed a copy number loss in a gene cluster in ECA 22:24,366,749–24,826,501 bp that includes Latherin (*LATH*) (**S1 Fig**). The reference genome assembly indicates just one copy of this gene, yet a copy-number gain was previously reported in a Quarter Horse using NGS data [7], and using array CGH a copy number loss was observed in the same region [31] although both were observed in the EquCab2.0 reference genome. *LATH* (also known as *BPIFA4*) is a member of the palate lung and nasal epithelium clone (*PLUNC*) family of proteins that is common in the oral cavity and saliva of mammals [33,34]. In horses this gene produces a surfactant protein that is expressed in the saliva and uniquely to the *Equidae*, as the primary protein component of sweat [35]. Therefore, equine Latherin protein may play an important role in mastication of fibrous food, as well as in enhancing evaporative cooling [33]. Therefore, it is reasonable to propose that the loss in *LATH* copies observed in this study is functional and could results from an evolutionary pressure affecting evaporative dissipation of heat, yielding athleticism and endurance in hot environments. We therefore sought to validate the presence of a copy number variation in the *LATH* gene and genes surrounding it using quantitative PCR (qPCR) analysis in eight horses, including the horse used to produce the EquCab2.0 and EquCab3.0 assembly. QPCR showed evidence of between two and six copies of *LATH* relative to a single copy control gene (*ASIP*) across seven individual horses of diverse breeds, although the limited resolution of the qPCR approach could not significantly differentiate copy numbers for individual animals when compared to the horse utilized in the reference genome assembly (**Figs 2 and S1**). Notably, the reference genome animal (“Twilight”) possess a mean copy number of four, by qPCR, though the assembly derived from her documents just one *LATH* copy. On the other hand, the qPCR analysis indicated statistically significant difference in copy number between horses of some nearby genes (*BPIFB4*, and *BPIFA1*) but not *BPIFA2* **(S1 Fig)**. Precise haplotype analysis of this complex CNV polymorphism is challenging due to the poor quality of the reference assembly within this region (unpublished data), and the technical challenges of qPCR result in limited resolution for this application. Thus, more precise determination of polymorphic CNVs and gene family expansion in the *LATH* region will require the use of more advanced techniques like long-read sequencing and digital qPCR quantification of the CNV in future work in order to definitively assert its nature [36]. Our annotation of the SVs indicated various types of translocations in all the horses overlapping the *LCORL* gene (chr3:107,527,197–107,548,838) known to affect variation in body size in horses (**S4 Table)** [37–39]. Our annotation of the SVs also indicated inversion events within the *ZFAT* gene (ECA chr9:77,041,542–77,245,704 bp, **S4 Table**) unique to the American Miniature horse (chr9: 33,399,941–78,068,077), Arabian horse (chr9: 3,507,891–81,594,291) and Percheron horse (chr9: 33,399,941–78,068,063). Moreover, our annotation showed an intrachromosomal translocation at (chr1: 7,107,280–52,656,577) overlapping the *ANKRD1* gene at chr1:37919690–37928431. ANKRD1 was previously implicated in affecting size variation in American Miniature horses [10].

#### Genome-wide diversity (π)

Nucleotide diversity (π) [40] is defined as the average number of nucleotide differences per site between two randomly chosen sequences in a population. Assessment of nucleotide diversity provides a valuable insight into the divergence of populations, inferring the demographic history of the species, as well as the historical size of the population [41]. Areas of lower than expected nucleotide diversity may signify signatures of past selection events [42]. Traditionally, such regions are found by comparing the same sequences from multiple individuals [40]. However, π as implemented in VCFtools is calculated from a single genome of a diploid individual [43–45]. In this study, regions with very low diversity were found by calculating π for each horse genome as the average number of differences between two chromosomes using 100 thousand bp non-overlapping windows in VCFtools [46]. This is not the ideal calculation of π as it typically requires multiple individuals from the same breed to indicate selection signatures. However, given that we have one genome per breed, it is a suitable approach.

The average nucleotide diversity across all six horses was 0.0017 for all SNP polymorphisms, and ranged between a minimum of 0.00001 and 0.0771 (**S9 Table**). Average diversity in the autosomal chromosomes for all horses was 0.0017, which is lower than the mean diversity of 0.00145 observed in the X chromosome (**S9 Table**). Since the X chromosome has three-quarters the effective population size (*N*_e_) of that of the autosomes, lower nucleotide diversity for the X chromosome is to be expected. However, a lower diversity level could also be due to a lower mutation rate (μ) on ECAX [47,48]. Clearly, X copy number differences specifically influence the calculation of nucleotide diversity levels in the male horses used in this study, as compared to the female reference animal (**Fig 1**).

Notably, the SNP dense regions on ECA20 and ECA12 were amongst the highest (top 1%) in nucleotide diversity (π) value (**Fig 1**). We used PANTHER (v14.0) [54] statistical over-representation test (using a Bonferroni correction at P < 0.05) analysis of xenoRefGene genes in these regions after removing exact duplicate gene names as horses have relatively few refseq genes. PANTHER further removed duplicate genes, keeping only a single gene ID cases at loci with two or more xenoRefGene names. The analysis revealed that enrichment for the T cell receptor signaling pathway and adaptive immune response (**S5 Table**). Paralogous regions like those in large gene families catalyze a collapse of these regions within computational genome assemblies leading to an inflation in the number of SNPs at these loci.

The bottom 1% of the empirical distribution of π values for each horse gives potential selected regions (**S8 Table**). PANTHER statistical over-representation test of genes in these regions (**S6 Table**) revealed genes involved in axonogenesis and synaptic transmission in the Arabian horse. In the Mangalarga Marchador horse the test revealed genes involved in negative regulation of heart process and regulation of insulin secretion and in the Tennessee Walking Horse, it revealed enrichment for the regulation of transcription and RNA biosynthesis. In the American Miniature horse, the test showed enrichment for genes involved in negative regulation of hormone secretion and of peptide transport and in the Percheron horse, it highlighted regions significantly enriched for genes involved in lipid catabolic processes and protein-containing complex disassembly. In the Native Mongolian Chakouyi horse, genes involved in glutamate receptor signaling pathway were overrepresented.

The low diversity regions found in this study did not overlap with the regions previously reported in Petersen *et al*. using Illumina SNP50 Beadchip and an F_ST_-based statistic [49], likely due to the differences in the genome version, sample size (744 horses from 33 breeds vs a single horse from 6 various breeds in this study) as well as the methodology. On the other hand, two gene regions reported in this study were also reported to be under selection in the horse by Orlando *et al*. [3]. However, unlike our study, Orlando *et al*. [3] aimed to detect selection signatures in modern horses and included several genomes and compared ancient horse genomes to those of Przewalski’s and modern domesticated horses. Namely, the three genes shared between both studies were *CHM* in the Arabian horse and *DENND1A* in the *COMMD1* in the Mongolian Chakouyi. *CHM* encodes a encodes a component of RAB geranylgeranyl transferase holoenzyme protein [50]. Mutations in this gene in humans are associated with retinal degeneration [50]. Overexpression of *DENND1A* produces a polycystic ovary syndrome theca phenotype [51].

## Materials and methods

### DNA collection and whole genome sequencing

All animal procedures for DNA sampling were approved by the Cornell University Institutional Animal Care and Use Committee (protocol 2008–0121). All horses were privately owned, and DNA was extracted either from 10mL of blood using Puregene whole-blood extraction kit (Qiagen Inc., Valencia, CA, USA) or hair samples (approximately 10 hair roots) using previously published methods following the owners’ permission [52]. Paired-end sequencing was performed at the Biotechnology Resource Center, Cornell University. For the construction of sequencing libraries, genomic DNA was sheared using a Covaris acoustic sonicator (Covaris, Woburn MA) and converted to Illumina sequencing libraries by blunt end-repair of the sheared DNA fragments, adenylation, ligation with paired-end adaptors, and enriched by PCR according to the manufacturer’s protocol (Illumina, San Diego CA). The size of the sequencing library was estimated by capillary electrophoresis using a Fragment Analyzer (AATI, Ames IA) and Qubit quantification (Life Technologies, Carlsbad CA). Cluster generation and paired-end sequencing on Illumina HiSeq instruments were performed according to the manufacturer’s protocols (Illumina, San Diego) at the Biotechnology Resource Center, Cornell University. The Percheron (PER), Miniature and Arabian horse (AMH) had a library read length of 100 bp and an average insert size of 188 bp, 181 bp and 181 bp respectively. The Brazilian Mangalarga Marchador (MM), a Native Mongolian Chakouyi (CH) and a Tennessee walking horse (TWH) had a library read length of 140 bp and an average insert size of 248 bp, 168 bp and 207 bp respectively.

### Read filtering and alignment

Raw reads were first inspected using the quality control program FastQC v10.1 (http://www.bioinformatics.babraham.ac.uk/projects/fastqc/). Then, the reads were quality filtered and adaptors removed using Trimmomatic [53]. The quality filtering utilized a sliding window of 4 bp and required a minimum mean Phred quality score of 20 within each window. Windows with an average quality less than 20 were sequentially removed from a read. Subsequently, reads with less than 60 bp of sequence remaining were removed from analysis along with their corresponding pairs. The genomes were then aligned to EquCab3 using BWA [54] using the *mem* procedure and the resulting output was converted to BAM format using Samtools *view* procedure. The BAM files were sorted using Samtools *sort* then, the duplicate reads in the BAM file were removed using the *MarkDuplicates* procedure in the Genome Analysis Toolkit (GATK) v 4.1.1.0 [55] procedures. After that, the *HaplotypeCaller* GATK procedure was used in GVCF mode to call SNPs and INDELs for each horse genome resulting in a GVCF file for each horse. The GVCF files were then combined using GATK *CombineGVCFs*. Finally, the SNPs and INDELs were jointly called using the GATK *GenotypeGVCFs* procedure.

### Identifying structural variations and copy number variations

Structural variations (SVs) and large INDELs were identified using SVDetect v1.2 using the default settings [56]. The program uses anomalously mapped read pairs to localize rearrangements within the genome and classify them into their various types. We first used the linking procedure to map all anomalous mapped paired-end reads were mapped onto the fragmented reference genome. We used a sliding window of size $2\mu +2\sqrt{2\sigma }$ to partition the genome, where *μ* is the estimated insert size and *σ* is the standard deviation. The length of steps in which the sliding window moved across the genome were set to half of the window size. After that, we used the filtering the procedure to filter out redundant links, using the default options and appropriate mean insert size value and standard deviation of the mapped mate-pairs (in bp) for each horse. Control-Freec v11.5 [57] was used to detect copy number variations (CNVs). The program uses GC-content and mapability profiles to normalize read count and therefore gives a better estimate of copy number profiles in high GC or low coverage regions [57]. A breakpoint threshold of 0.6 and a coefficient of variation of 0.05 were used in the analysis.

### Variant annotation

We used SNPEff v4.3 [58] to annotate the SNPs and short INDELs using the latest available gene annotation database (EquCab3.0). The output of SNPEff is a full list of effects per variant. SNPs and Indels located within 5,000 bases (5 kb) upstream or downstream genes as well as those within exons, introns, splice sites, and 5’ and 3’ untranslated regions (UTRs) were also annotated. Since SNPEff output can be integrated into GATK VCF file, we have produced an annotated version of the GATK VCF file which can be loaded and viewed easily in genome browsers. The CNVs and SV breakpoints overlapping xenoRefGene genes were detected using Bedtools (v2.23.0).

We used Nucleotide Diversity (π) to identify regions with low diversity. For each of the genomes, the nucleotide diversity (π) was calculated for the SNPs in 100 thousand bp non-overlapping windows using VCFtools v1.10 using the command *window-pi* [46]. Regions in the lower 1% tail of the π distribution were considered low diversity regions following a similar approach by Branca *et al*. [59]. Genes in these regions were annotated for biological process, using PANTHER v14.1 [60]. Circos plots [61] summarizing the distribution of the genomic variations were then created for each of the genomes and a summary circos plot was created to highlight variants in common between the six genomes. To enhance visualization, we removed the small intrachromosomal elements (endpoints size <10 bp) and interchromosomal elements (endpoints size <500 bp) due to their abundance in the output which makes it difficult to visualize in the circos plot.

### RT q-PCR analysis of the CNV near Latherin gene

We used Quantitative PCR to quantify the copy number variation in exons of 4 genes from 8 horses, including the horse used to produce the EquCab 3.0 reference genome assembly. Primers designed in Primer3 v0.4.0 targeted exons overlapping the NGS identified copy number variation [62] (**Table 3**). Genomic DNA (25 ng) was amplified in 10 uL reactions using the Quanta Biosciences PerfeCta SYBR Green (FastMix) as per the manufacturers recommended conditions (Gaithersburg, MD, USA). *ASIP* exon 2 was amplified as reference single-copy gene. Thermocycling and detection were performed using PCR on the Illumina Eco Real-Time PCR System using parameters recommended for the Quanta Mix (58°C annealing). Copy numbers were calculated relative to the reference genome horse. We substituted the Percheron and American Miniature horses by horses from the same breed, as DNA samples from the original two sequenced horses were unavailable.

**Table 3:** Real-time quantitative PCR primers.

Gene	Forward primer	Reverse primer	Amplicon size (BP)
*LATH*	AGGACTCCTTGACGGGAACT	AGGGCCAACCAAGATGTTC	112
*BPIFA1*	GGAGAAGCACTCACCAGCTC	CTCCAGAGTTCCCGTTTCCT	207
*BPIFB4*	TGTTGGTGGTGTTCCCTACA	TAGTCGCCATTTCGAAGGTC	198
*BPIFA2*	CGTTTTTGTCAGGTGTCTTCC	CCCAAAGAACCATCCACAGT	157

## Supporting information

S1 Table Annotation of SNPs and INDELs by position and putative functional consequence. (DOCX)

S2 Table Annotations of copy number variants (CNVs) detected in six horses. xenoRefGene genes names overlapping the variants locations are given.
(XLSX)

S3 Table Number of copy number variants (CNVs) gains and loss detected in six horses. (XLSX)

S4 Table Structural variants (SVs) detected in six horses and genes overlapping the variants locations are given for each horse. (XLSX)

S5 Table Statistical overrepresentation test (Bonferroni-corrected for P < 0.05) for genes in high π regions in the six horses in chromosomes 12 and 20. (DOCX)

S6 Table Top five categories for statistical overrepresentation test for genes in low π regions in various horses. (DOCX)

S7 Table Shared copy number variants between the current study and Solé et al., 2019 current study CNVs are in columns A-G, whereas CNVs in Solé et al., 2019 study are in columns H-L. (XLSX)

S8 Table Chromosomal regions and genes in low nucleotide diversity (π) regions (lower 1% of the empirical distribution) detected in the six horses genomes. (XLSX)

S9 Table Nucleotide diversity (π) estimates from 100 thousand bp non-overlapping windows in the six horses genomes. (XLSX)

S10 Table Links to SNPs, INDELS as well as SVs and CNVs tracks in CyVerse. (TXT)

S1 Fig Screen shot of EquCab3.0 chr22:24,366,749–24,826,501 showing the CNVs around *LATH* gene. (TIF)
