# Supplementary material for: Whole genome detection of sequence and structural polymorphism in six diverse horses
Source: PLoS One. 2020 Apr 9;15(4):e0230899. doi: 10.1371/journal.pone.0230899 (PMC7144971; doi:10.1371/journal.pone.0230899)
Supplement: S1 Table — (DOCX) [file pone.0230899.s001.docx]

|  | **SNPs** |  | **INDELs** |  |
| --- | --- | --- | --- | --- |
| **Effects according to region** | **Count** | **Percentage** | **Count** | **Percentage** |
| Downstream | 1,870,705 | 5.62% | 233,726 | 5.70% |
| Exon | 489,808 | 1.47% | 28,473 | 0.69% |
| Intron | 9,009,277 | 27.06% | 1,079,528 | 26.31% |
| Intergenic | 19,917,885 | 59.83% | 2,517,407 | 61.35% |
| Splice Site Acceptor | 1,891 | 0.01% | 334 | 0.01% |
| Splice Site Donor | 2,190 | 0.01% | 402 | 0.01% |
| Transcript | 1,926 | 0.01% | 334 | 0.01% |
| Upstream | 1,879,171 | 5.65% | 230,236 | 5.61% |
| 3’ UTR | 73,988 | 0.222% | 9,450 | 0.23% |
| 5’ UTR | 41,457 | 0.125% | 3,462 | 0.084% |

S1 Table: Annotation of SNPs and INDELs by position and putative functional consequence.
