# Supplementary material for: Whole genome detection of sequence and structural polymorphism in six diverse horses
Source: PLoS One. 2020 Apr 9;15(4):e0230899. doi: 10.1371/journal.pone.0230899 (PMC7144971; doi:10.1371/journal.pone.0230899)
Supplement: S5 Table — (DOCX) [file pone.0230899.s005.docx]

**S5 Table**: Statistical overrepresentation test (Bonferroni-corrected for P < 0.05) for genes in high π regions in the six horses in chromosomes 12 and 20.

| **GO biological process complete** | **Number** | **Expected** | **Fold Enrichment** | **+/-** | **P value** |
| --- | --- | --- | --- | --- | --- |

| T cell receptor signaling pathway | [25](http://www.pantherdb.org/tools/gxIdsList.do?acc=GO:0050852&reflist=1) | [1](http://www.pantherdb.org/tools/gxIdsList.do?acc=GO:0050852&list=Client%20Text%20Box%20Input&organism=Equus%20caballus) | 54.66 | + | 1.89E-02 |
| --- | --- | --- | --- | --- | --- |
| cell surface receptor signaling pathway involved in cell-cell signaling | [45](http://www.pantherdb.org/tools/gxIdsList.do?acc=GO:1905114&reflist=1) | [1](http://www.pantherdb.org/tools/gxIdsList.do?acc=GO:1905114&list=Client%20Text%20Box%20Input&organism=Equus%20caballus) | 30.37 | + | 3.31E-02 |
| antigen receptor-mediated signaling pathway | [55](http://www.pantherdb.org/tools/gxIdsList.do?acc=GO:0050851&reflist=1) | [1](http://www.pantherdb.org/tools/gxIdsList.do?acc=GO:0050851&list=Client%20Text%20Box%20Input&organism=Equus%20caballus) | 24.84 | + | 4.02E-02 |
| immune response-activating cell surface receptor signaling pathway | [63](http://www.pantherdb.org/tools/gxIdsList.do?acc=GO:0002429&reflist=1) | [1](http://www.pantherdb.org/tools/gxIdsList.do?acc=GO:0002429&list=Client%20Text%20Box%20Input&organism=Equus%20caballus) | 21.69 | + | 4.58E-02 |
| immune response-regulating cell surface receptor signaling pathway | [63](http://www.pantherdb.org/tools/gxIdsList.do?acc=GO:0002768&reflist=1) | [1](http://www.pantherdb.org/tools/gxIdsList.do?acc=GO:0002768&list=Client%20Text%20Box%20Input&organism=Equus%20caballus) | 21.69 | + | 4.58E-02 |
| regulation of membrane potential | [130](http://www.pantherdb.org/tools/gxIdsList.do?acc=GO:0042391&reflist=1) | [1](http://www.pantherdb.org/tools/gxIdsList.do?acc=GO:0042391&list=Client%20Text%20Box%20Input&organism=Equus%20caballus) | 10.51 | + | 9.17E-02 |
