# Supplementary material for: Whole genome detection of sequence and structural polymorphism in six diverse horses
Source: PLoS One. 2020 Apr 9;15(4):e0230899. doi: 10.1371/journal.pone.0230899 (PMC7144971; doi:10.1371/journal.pone.0230899)
Supplement: S6 Table — (DOCX) [file pone.0230899.s006.docx]

**S6 Table**: Top five categories for Statistical overrepresentation test for genes in low π regions in various horses.

**ARB Horse:**

| **PANTHER GO-Slim Biological Process** | **number** | **expected** | **Fold Enrichment** | **+/-** | **P value** |
| --- | --- | --- | --- | --- | --- |
| regulation of leukocyte migration | 7 | 2 | 61 | + | 7.58E-04 |
| synaptic transmission, glutamatergic | 40 | 3 | 16.01 | + | 1.06E-03 |
| axonogenesis | 96 | 4 | 8.9 | + | 1.24E-03 |
| lamellipodium assembly | 10 | 2 | 42.7 | + | 1.38E-03 |
| cell surface receptor signaling pathway involved in cell-cell signaling | 45 | 3 | 14.23 | + | 1.46E-03 |

**PER Horse:**

| **PANTHER GO-Slim Biological Process** | **number** | **expected** | **Fold Enrichment** | **+/-** | **P value** |
| --- | --- | --- | --- | --- | --- |
| protein depolymerization | 22 | 3 | 25.41 | + | 3.12E-04 |
| cellular protein complex disassembly | 24 | 3 | 23.29 | + | 3.94E-04 |
| regulation of ion transport | 71 | 4 | 10.5 | + | 6.98E-04 |
| lipid catabolic process | 8 | 2 | 46.58 | + | 1.24E-03 |
| protein-containing complex disassembly | 37 | 3 | 15.11 | + | 1.27E-03 |

**CH Horse:**

| **PANTHER GO-Slim Biological Process** | **number** | **expected** | **Fold Enrichment** | **+/-** | **P value** |
| --- | --- | --- | --- | --- | --- |
| glutamate receptor signaling pathway | 35 | 4 | 32.53 | + | 1.48E-02 |
| biological regulation | 2816 | 25 | 2.53 | + | 8.67E-03 |
| synaptic transmission, glutamatergic | 40 | 4 | 28.47 | + | 2.42E-02 |
| cell surface receptor signaling pathway involved in cell-cell signaling | 45 | 4 | 25.3 | + | 3.72E-02 |
| regulation of biological quality | 609 | 12 | 5.61 | + | 2.17E-03 |

**MM Horse:**

| **PANTHER GO-Slim Biological Process** | **number** | **expected** | **Fold Enrichment** | **+/-** | **P value** |
| --- | --- | --- | --- | --- | --- |
| heart process | 2 | 2 | > 100 | + | 1.38E-01 |
| negative regulation of hormone secretion | 3 | 2 | > 100 | + | 2.29E-01 |
| regulation of ion transport | 71 | 4 | 1.39E+01 | + | 3.45E-01 |
| negative regulation of protein transport | 5 | 2 | 9.88E+01 | + | 4.78E-01 |
| regulation of insulin secretion | 5 | 2 | 98.78 | + | 4.78E-01 |

**AMH Horse:**

| **PANTHER GO-Slim Biological Process** | **number** | **expected** | **Fold Enrichment** | **+/-** | **P value** |
| --- | --- | --- | --- | --- | --- |
| negative regulation of hormone secretion | 3 | 2 | > 100 | + | 9.68E-02 |
| negative regulation of cell communication | 5 | 2 | > 100 | + | 2.03E-01 |
| negative regulation of protein transport | 5 | 2 | > 100 | + | 2.03E-01 |
| peptide secretion | 8 | 2 | 94.89 | + | 4.32E-01 |
| regulation of hormone secretion | 8 | 2 | 94.89 | + | 4.32E-01 |

**TWH Horse**

| **PANTHER GO-Slim Biological Process** | **number** | **expected** | **Fold Enrichment** | **+/-** | **P value** |
| --- | --- | --- | --- | --- | --- |
| regulation of transcription, DNA-templated | 1833 | 21 | 2.13E+00 | + | 1.10E-03 |
| regulation of nucleic acid-templated transcription | 1882 | 21 | 2.08E+00 | + | 1.30E-03 |
| regulation of RNA biosynthetic process | 1884 | 21 | 2.08E+00 | + | 1.31E-03 |
| axon guidance | 87 | 4 | 8.57E+00 | + | 1.44E-03 |
| neuron projection guidance | 90 | 4 | 8.28 | + | 1.62E-03 |
