# Supplementary figures and images for: Whole genome detection of sequence and structural polymorphism in six diverse horses
Source: PLoS One. 2020 Apr 9;15(4):e0230899. doi: 10.1371/journal.pone.0230899 (PMC7144971; doi:10.1371/journal.pone.0230899)

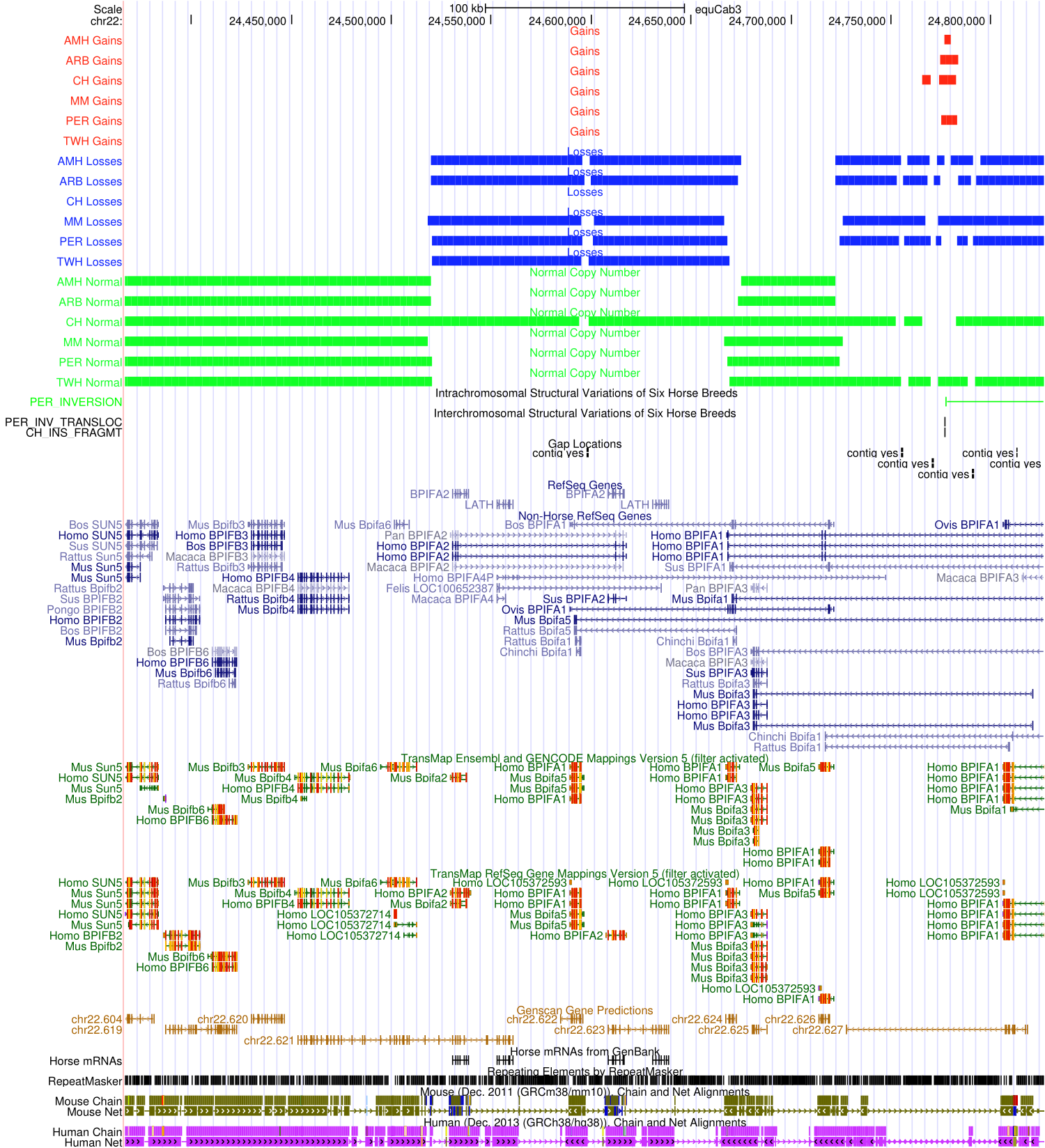

Supplement: S1 Fig — (TIF) [file pone.0230899.s011.tif]
